# The Outcome of Sutureless in Partial Nephrectomy: A Systematic Review and Meta-Analysis

**DOI:** 10.1155/2022/5260131

**Published:** 2022-09-22

**Authors:** Peng Liu, Yan Li, Benkang Shi, Qiujie Zhang, Hu Guo

**Affiliations:** Qilu Hospital of Shandong University, Jinan, China

## Abstract

**Purpose:**

To compare the effect of sutureless versus standard suture (double-layer suture) during renorrhaphy in laparoscopic or robotic-assisted partial nephrectomy on perioperative and renal function outcomes.

**Methods:**

PubMed, Embase, and other sources were searched for randomized controlled trials or retrospective studies comparing sutureless partial nephrectomy versus standard suture partial nephrectomy. A systematic review and meta-analysis were performed by two reviewers independently.

**Results:**

Five retrospective studies were included with a total of 634 patients. The results showed that there was a significant difference in the decline of estimated glomerular filtration rate (*I*^2^ = 98.5%; WMD, -4.19 ml/min; 95% CI, -7.64 to -0.73; *P* < 0.001) and no significant difference in postoperative complications (*I*^2^ = 0; RR, 1.31; 95% CI, 0.61 to 2.81; *P* = 0.623). A significant advantage in terms of operating time (*I*^2^ = 53.9%; WMD, -29.08 min; 95% CI, -33.06 to -25.10; *P* = 0.069) and warm ischemia time (*I*^2^ = 38.5%; WMD, -6.17 min; 95% CI, -6.99 to -5.36; *P* = 0.165) favored sutureless, while there was no significant difference in blood loss (*I*^2^ = 58.1%; WMD, 3.10 ml; 95% CI, -39.18 to 45.38; *P* = 0.049).

**Conclusion:**

Sutureless during renorrhaphy is feasible and safe compared with standard suture. Sutureless can shorten the operating time and warm ischemia time without increasing postoperative complications, and thus, it protects renal function.

## 1. Introduction

Renal cell carcinoma accounts for about 2% of all cancers [1], and the detection rate of kidney cancer is getting higher and higher with the development of diagnosis technique. Partial nephrectomy is recommended for small renal tumors because of better renal functional prognosis, compared to radical nephrectomy [2, 3]. Following rapid technical advances, laparoscopic and robotic minimally invasive techniques have become the mainstream. Traditional partial nephrectomy performs a double-layer suture (inner and cortical) during renorrhaphy [4–6], known as standard suture, which may damage renal vessels, reduce renal parenchyma, and increase warm ischemia time resulting in renal dysfunction [7]. Recently, there has been a growing application of sutureless during renorrhaphy in laparoscopic and robotic-assisted partial nephrectomy [8–10]. Sutureless uses various hemostatic materials and surgical equipment instead of suturing during renorrhaphy to achieve the purpose of hemostasis [11, 12]. The sutureless technique is simpler, has shorter operating time and less parenchymal damage than the standard suture, and may result in better perioperative outcomes and less renal function impairment. Indeed, two previous meta-analyses reported that single-layer suture versus double-layer suture did have better perioperative outcomes and less renal function loss [5, 6], so it is reasonable to believe that sutureless will be a worthwhile surgical technique to develop. A recent meta-analysis was conducted, which compared suture and sutureless during renorrhaphy [13]. However, the definition of suture was not clear in this paper, including single-layer suture and double-layer suture, which may increase heterogeneity and lead to inaccurate results. Moreover, the limited included studies of this meta-analysis heavily decreased the convening and applicability of the conclusion.

Therefore, it was necessary to compare the effect of sutureless versus standard suture in partial nephrectomy on perioperative and renal function outcomes.

## 2. Methods

This meta-analysis was conducted on the basis of the Preferred Reporting Items for Systematic Reviews and Meta-Analyses (PRISMA) statement for meta-analysis [14]. This study was registered with PROSPERO (CRD42022293977).

### 2.1. Data Sources and Searches

PubMed, Embase, and other sources were searched to find relevant articles up to 18 September 2021. The detailed search strategy is shown in Supplemental Table S1. In addition to electronic databases, we also searched conference abstracts, key reviews, book chapters, and international trial registers manually.

### 2.2. Eligibility Criteria

Studies comparing double-layer suture partial nephrectomy and sutureless partial nephrectomy were included. The detailed eligibility criteria were summarized using a specific population (P), intervention (I), comparator (C), outcome (O), and study design (S) (PICOS) framework (Supplemental Table S2). The title and abstract of the article were first reviewed by two reviewers (PL and YL) to determine their suitability for inclusion. Then, a more comprehensive assessment was made by looking at the full text to determine whether it should be included in the study. Any discrepancies were settled by discussion with the third reviewer (HG).

### 2.3. Data Extraction

Two reviewers (PL, YL) independently extracted data using a standardized extraction form, and the differences were resolved by another reviewer (HG). The primary outcome was mean change in the renal function, denoted by the decline of estimated glomerular filtration rate. The secondary outcomes were perioperative outcomes including operating time, warm ischemia time, blood loss, and postoperative complication. For studies reporting medians and ranges [or interquartile ranges (IQR)], the validated mathematical model [15, 16] was used to convert the median (range or IQR) to mean [standard deviation (SD)]. Main characteristics of qualified studies such as age, tumor size, baseline renal function, and sutureless technique were extracted to conduct further analysis.

### 2.4. Risk of Bias Assessment

Risk of bias assessment was performed according to the Newcastle–Ottawa scale (NOS) for nonrandomized trials. Two independent reviewers (PL and YL) assessed the risk of bias in all included studies according to NOS. Any inconsistency was discussed and resolved by HG to reach an agreement.

### 2.5. Statistical Analysis

For continuous outcomes, the weighted mean difference (WMD) was used as a summary measure, whereas the risk ratio (RR) with 95% confidence interval (CI) was calculated for binary variables. Statistical heterogeneity between studies included was assessed using *I*^2^. Pooled estimates were calculated using a fixed-effects model if a low level of heterogeneity between the studies (*I*^2^ < 50%) was identified; otherwise, a random-effects model was used when a high level of heterogeneity was detected. Sensitivity analysis was performed in analyses with high heterogeneity to assess the robustness of the findings. A *P* value < 0.05 was considered statistically significant. All statistical analyses were performed using STATA16.0 (College Station, Texas, USA).

## 3. Results

**Figure 1:**
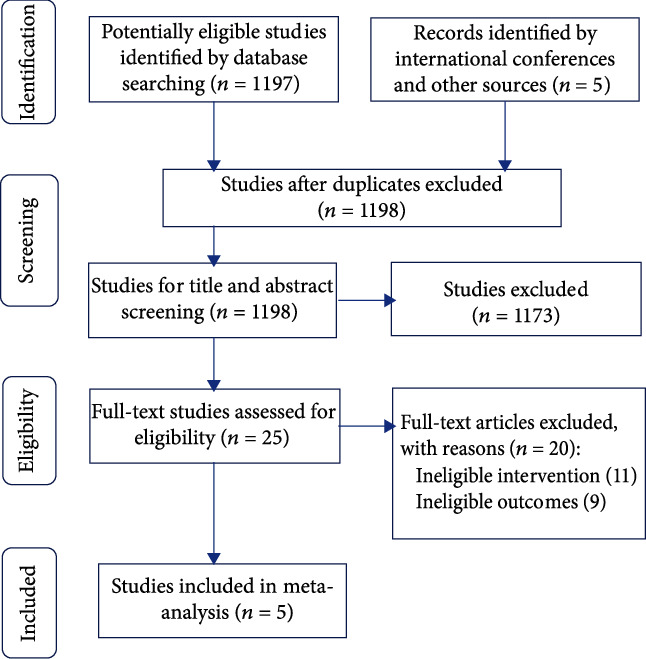
Study selection.

The literature search identified 1198 unique studies, and 1173 studies were excluded during screening (Figure 1). Of the 25 full-text articles assessed for eligibility, 11 were excluded for lack of useful data and 9 were excluded for ineligible intervention. Overall, 5 studies [8, 9, 11, 12, 17], which included 634 patients, were involved in this meta-analysis. Summaries of all included studies are shown in Table 1. The two groups analyzed were comparable in terms of age, preoperative glomerular filtration rate, and tumor size. Risk of bias assessment is summarized in Table 1.

**Figure 2:**
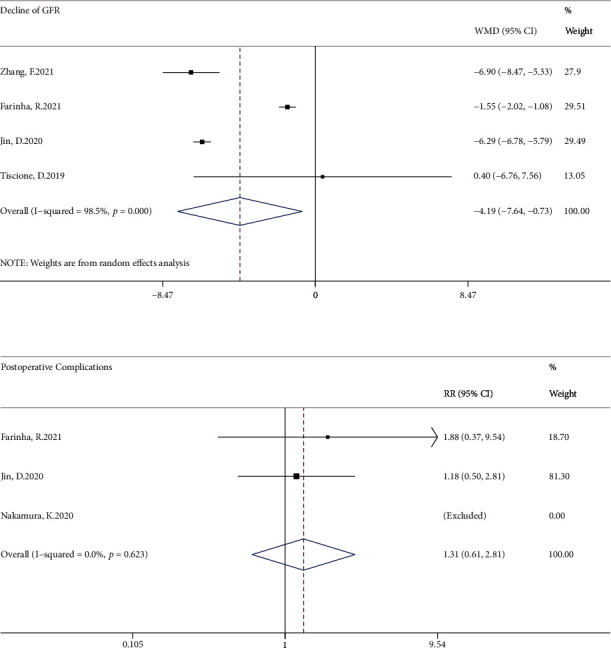
Forest plots of the decline of estimated glomerular filtration rate and postoperative complications for sutureless versus double-layer suture partial nephrectomy. GFR: glomerular filtration rate; WMD: weighted mean difference; RR: relative risk.

**Figure 3:**
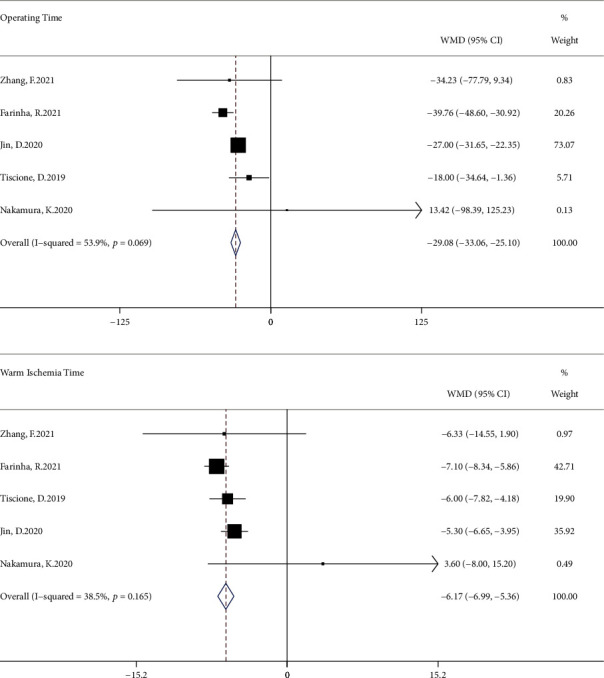
Forest plots of the decline of operating time and warm ischemia time for sutureless versus double-layer suture partial nephrectomy. WMD: weighted mean difference.

There was a significant difference in the decline of estimated glomerular filtration rate (*I*^2^ = 98.5%; WMD, -4.19 ml/min; 95% CI, -7.64 to -0.73; *P* < 0.001) and no significant difference in postoperative complications (*I*^2^ = 0; RR, 1.31; 95% CI, 0.61 to 2.81; *P* = 0.623; Figure 2). A significant advantage in terms of operating time (*I*^2^ = 53.9%; WMD, -29.08 min; 95% CI, -33.06 to -25.10; *P* = 0.069) and warm ischemia time (*I*^2^ = 38.5%; WMD, -6.17 min; 95% CI, -6.99 to -5.36; *P* = 0.165) favored sutureless (Figure 3), while there was no significant difference in blood loss (*I*^2^ = 58.1%; WMD, 3.10 ml; 95% CI, -39.18 to 45.38; *P* = 0.049; Supplementary Figure S1).

Sensitivity analysis was performed in analyses with high heterogeneity (the decline of estimated glomerular filtration rate, operating time, and blood loss). After excluding the study by Farinha et al. [8], the heterogeneity of the decline of estimated glomerular filtration rate and operating time decreased significantly, and *I*^2^ < 50%; however, the results were still consistent with previous analyses (Supplementary Figure S2 and S3). Despite heterogeneity being significantly reduced after excluding the study by Tiscione et al. [11] in terms of blood loss, the result was also altered, with sutureless significantly reducing blood loss, compared to the double-layer suture (*I*^2^ = 0; WMD, -20.94 ml; 95% CI, -41.43 to -0.44; *P* = 0.592) (Supplementary Figure S4).

## 4. Discussion

With the development of minimally invasive surgical technology, laparoscopic or robotic-assisted partial nephrectomy has become the mainstream of small renal tumor treatment [18]. After resection of the mass, renorrhaphy was performed using a double-layer suture technique. In recent years, sutureless technology appears in laparoscopic or robotic-assisted partial nephrectomy with the development of hemostatic materials and equipment, which reduced the difficulty of partial nephrectomy and shortened the operating time.

This meta-analysis revealed that sutureless during renorrhaphy in laparoscopic or robotic-assisted partial nephrectomy could better preserve renal function loss compared with the double-layer suture.

Anceschi et al. recently introduced a novel composite trifecta outcome, with the ability to predict both oncologic and functional endpoints of PN. This novel trifecta allows to regulate surgical training, introduce technical innovations, and describe their outcomes with a simple and univocal terminology and should be adopted to compare surgical approaches.

In terms of perioperative outcomes, sutureless partial nephrectomy reduced operative time and warm ischemia time obviously, which may be one of the reasons why it protects renal function [19–21]. In contrast to the results of the primary analysis, the results of sensitivity analysis suggested that sutureless can reduce the amount of blood loss compared with the double-layer suture, which needs further investigation. The most concerning aspect of sutureless during renorrhaphy is postoperative complications, especially bleeding and urinary leakage [22]. This meta-analysis revealed that there was no significant difference between the double-layer suture and sutureless in postoperative complications. With the development of sutureless and hemostatic technology, the probability of urinary leakage and bleeding may be further reduced. Since the renal parenchyma is not sutured during renorrhaphy and the renal vessels are not damaged, sutureless greatly reduces the incidence of pseudoaneurysm [23]. Furthermore, Ferriero et al. recently described a surgical technique and assessed the safety and oncologic and functional outcomes of a single center experience of sutureless, off-clamp robotic partial nephrectomy [24]. These results suggest that sutureless partial nephrectomy could be technically feasible and safe, yielding acceptable perioperative and renal function outcomes. Anceschi et al. recently introduced a novel composite trifecta outcome, with the ability to predict both oncologic and functional endpoints of PN [25]. This novel trifecta allows to regulate surgical training, introduce technical innovations, and describe their outcomes with a simple and univocal terminology and should be adopted to compare surgical approaches. Therefore, we look forward to more studies to evaluate sutureless during renorrhaphy in laparoscopic or robotic-assisted partial nephrectomy on perioperative and renal function outcomes through this novel composite trifecta to verify the results of this article.

There were several limitations to this meta-analysis study. The studies included in this meta-analysis were retrospective, and the number of studies is insufficient, especially regarding postoperative complications. Therefore, more well-designed randomized controlled trials are needed to further evaluate the two suture techniques during renorrhaphy in laparoscopic or robotic-assisted partial nephrectomy.

## 5. Conclusion

This meta-analysis suggested that sutureless during renorrhaphy could be feasible and safe, compared to standard suture. Sutureless can shorten the operating time and warm ischemia time without increasing postoperative complications and better protect renal function. Sutureless is worth further development because of its simple operation and easy learning.

## Figures and Tables

**Table 1:** Characteristics of included studies in the review.

Author, year (ref.)	T stage	Study design	Renorrhaphy technique	Sutureless technique	Outcomes	SQ
Zhang, F., 2021 [17]	T1	RTP, PSM	Sutureless (*n* = 116) Double-layer suture (*n* = 116)	Monopolar coagulation; NBCA	Surgical approach, OT, WIT, EBL, positive, surgical margin, length of stay, postoperative complications, eGFR	6
Farinha, R., 2021 [8]	T1–2	RTP, PSM	Sutureless (*n* = 29) Double-layer suture (*n* = 29)	Bipolar or monopolar coagulation; hemostatic agents	OT, WIT, EBL, length of stay, postoperative complications, eGFR, AKI	9
Jin, D., 2020 [9]	T1a	RTP, PSM	Sutureless (*n* = 65) Double-layer suture (*n* = 189)	Monopolar coagulation; hemostatic agents	OT, WIT, EBL, conversion to nephrectomy, length of stay, postoperative complications, eGFR, AKI	8
Tiscione, D., 2019 [11]	T1–2	RTP	Sutureless (*n* = 19) Double-layer suture (*n* = 21)	Fibrin glue	OT, WIT, EBL, length of stay, postoperative complications, eGFR, pathologic and follow-up findings	6
Nakamura, K., 2020 [12]	T1	RTP, PSM	Sutureless (*n* = 25) Double-layer suture (*n* = 25)	Soft COAG	OT, WIT, EBL, eGFR, positive surgical margin	6

PSM: propensity score matching; RTP: retrospective; OT: operating time; WIT: warm ischemia time; EBL: estimated blood loss; eGFR: estimated glomerular filtration rate; NBCA: *N*-butyl-2-cyanoacrylate; AKI: acute kidney injury; SQ: study quality according to the Newcastle–Ottawa scale.

## Data Availability

No additional data are available.

## Supplementary Materials

Supplementary Materials Table S1: search strategy. Table S2: eligibility criteria. Figure S1: forest plots of blood loss for sutureless versus double-layer suture partial nephrectomy. Figure S2: sensitivity analysis of estimated glomerular filtration rate. Figure S3: sensitivity analysis of operating time. Figure S4: sensitivity analysis of blood loss.
