# Supplementary material for: The Outcome of Sutureless in Partial Nephrectomy: A Systematic Review and Meta-Analysis
Source: Biomed Res Int. 2022 Sep 22;2022:5260131. doi: 10.1155/2022/5260131 (PMC9526602; doi:10.1155/2022/5260131)
Supplement: Supplementary Materials — Table S1: search strategy. Table S2: eligibility criteria. Figure S1: forest plots of blood loss for sutureless versus double-layer suture partial nephrectomy. Figure S2: sensitivity analysis of estimated glomerular filtration rate. Figure S3: sensitivity analysis of operating time. Figure S4: sensitivity analysis of blood loss. [file 5260131.f1.zip › Supplementary Information.docx]

Sutureless during partial nephrectomy may modify perioperative and renal function outcomes: a systematic review and meta-analysis

Peng Liu^1^, Yan Li^1^, Benkang Shi^1^, Qiujie Zhang^1^ and Hu Guo^1^

Correspondence should be addressed to guohu97@sdu.edu.cn

1 Qilu Hospital of Shandong University, Jinan, China

**Table S1.** **Search strategy**

**1. Search strategy on PubMed**

(((((((((((((((((((((((((((Robotic Surgical Procedures) OR (Procedure, Robotic Surgical)) OR (Robotic Surgical Procedure)) OR (Robot Surgery)) OR (Robot Surgeries)) OR (Surgery, Robot)) OR (Robot-Assisted Surgery)) OR (Robot Assisted Surgery)) OR (Robot-Assisted Surgeries)) OR (Surgery, Robot-Assisted)) OR (Robot-Enhanced Procedures)) OR (Procedure, Robot-Enhanced)) OR (Robot Enhanced Procedures)) OR (Robot-Enhanced Procedure)) OR (Robotic-Assisted Surgery)) OR (Surgical Procedures, Robotic)) OR (Surgical Procedure, Robotic)) OR (Robotic-Assisted Surgeries)) OR (Surgery, Robotic-Assisted)) OR (Robot-Enhanced Surgery)) OR (Robot Enhanced Surgery)) OR (Robot-Enhanced Surgeries)) OR (Surgery, Robot-Enhanced)) OR (Robotic Assisted Surgery)) OR (Procedures, Robotic Surgical)) OR ((((((((((((((((((((Laparoscopy) OR (Laparoscopies)) OR (Celioscopy)) OR (Celioscopies)) OR (Peritoneoscopy)) OR (Peritoneoscopies)) OR (Surgical Procedures, Laparoscopic)) OR (Laparoscopic Surgical Procedure)) OR (Procedure, Laparoscopic Surgical)) OR (Surgery, Laparoscopic)) OR (Laparoscopic Surgery)) OR (Laparoscopic Surgeries)) OR (Surgeries, Laparoscopic)) OR (Laparoscopic Assisted Surgery)) OR (Surgeries, Laparoscopic Assisted)) OR (Surgery, Laparoscopic Assisted)) OR (Laparoscopic Assisted Surgeries)) OR (Laparoscopic Surgical Procedures)) OR (Procedures, Laparoscopic Surgical)) OR (Surgical Procedure, Laparoscopic))) AND (((((partial nephrectomy) OR (nephron-sparing surgery)) OR (conservative surgery)) OR (enucleation)) OR (enucleoresection))) AND (renorrhaphy OR sutur* OR reconstruction)

**2. Search strategy on Embase**

('partial nephrectomy'/exp OR 'nephron-sparing surgery':ab,ti OR 'conservative surgery':ab,ti OR enucleation:ab,ti OR enucleoresection:ab,ti) AND ('laparoscopy'/exp OR laparoscopies:ab,ti OR celioscopy:ab,ti OR celioscopies:ab,ti OR peritoneoscopy:ab,ti OR peritoneoscopies:ab,ti OR 'surgical procedures, laparoscopic':ab,ti OR 'laparoscopic surgical procedure':ab,ti OR 'procedure, laparoscopic surgical':ab,ti OR 'procedures, laparoscopic surgical':ab,ti OR 'surgery, laparoscopic':ab,ti OR 'laparoscopic surgical procedures':ab,ti OR 'laparoscopic surgery':ab,ti OR 'laparoscopic surgeries':ab,ti OR 'surgeries, laparoscopic':ab,ti OR 'laparoscopic assisted surgery':ab,ti OR 'laparoscopic assisted surgeries':ab,ti OR 'surgeries, laparoscopic assisted':ab,ti OR 'surgery, laparoscopic assisted':ab,ti OR 'surgical procedure, laparoscopic':ab,ti OR 'robot assisted surgery'/exp OR 'procedure, robotic surgical':ab,ti OR 'procedures, robotic surgical':ab,ti OR 'robotic surgical procedure':ab,ti OR 'surgical procedure, robotic':ab,ti OR 'robot surgery':ab,ti OR 'robot surgeries':ab,ti OR 'surgery, robot':ab,ti OR 'robot-assisted surgery':ab,ti OR 'robot assisted surgery':ab,ti OR 'robot-assisted surgeries':ab,ti OR 'surgery, robot-assisted':ab,ti OR 'robot-enhanced procedures':ab,ti OR 'procedure, robot-enhanced':ab,ti OR 'robot enhanced procedures':ab,ti OR 'robot-enhanced procedure':ab,ti OR 'surgical procedures, robotic':ab,ti OR 'robotic-assisted surgery':ab,ti OR 'robotic assisted surgery':ab,ti OR 'robotic-assisted surgeries':ab,ti OR 'surgery, robotic-assisted':ab,ti OR 'robot-enhanced surgery':ab,ti OR 'robot enhanced surgery':ab,ti OR 'robot-enhanced surgeries':ab,ti OR 'surgery, robot-enhanced':ab,ti) AND ('suture'/exp OR suture:ab,ti OR 'staple, surgical':ab,ti OR 'staples, surgical':ab,ti OR 'surgical staple':ab,ti OR 'surgical staples':ab,ti OR renorrhaphy:ab,ti OR reconstruction:ab,ti OR sutur*:ab,ti)

**Table S2. eligibility criteria**

|  | **Inclusion criteria** | **Exclusion criteria** |
| --- | --- | --- |
| Patient population | Patients >18 years with renal masses, undergoing LPN or RAPN | Patients＜18 years; open partial nephrectomy |
| Interventions | Sutureless during renorrhaphy | Single-layer suture or other suture techniques during renorrhaphy |
| Comparator | Double-layer suture (inner and cortical) | Single-layer suture or other suture techniques during renorrhaphy |
| Outcomes | Renal functional outcomes: decline of eGFR  Intra- or perioperative outcomes: OT; WIT; BL; postoperative complication |  |
| Study design | RCT or retrospective study | Case reports, abstract meetings, book chapters, editorials and previous reviews on the topic |

LPN, laparoscopic partial nephrectomy; RAPN, robot-assisted partial nephrectomy; RCT, randomized controlled trail; OT, operating time; WIT, warm ischemia time; EBL, estimated blood loss; eGFR, estimated glomerular filtration rate.

**Figure S1. Forest plots of blood loss for sutureless versus double-layer suture partial nephrectomy**


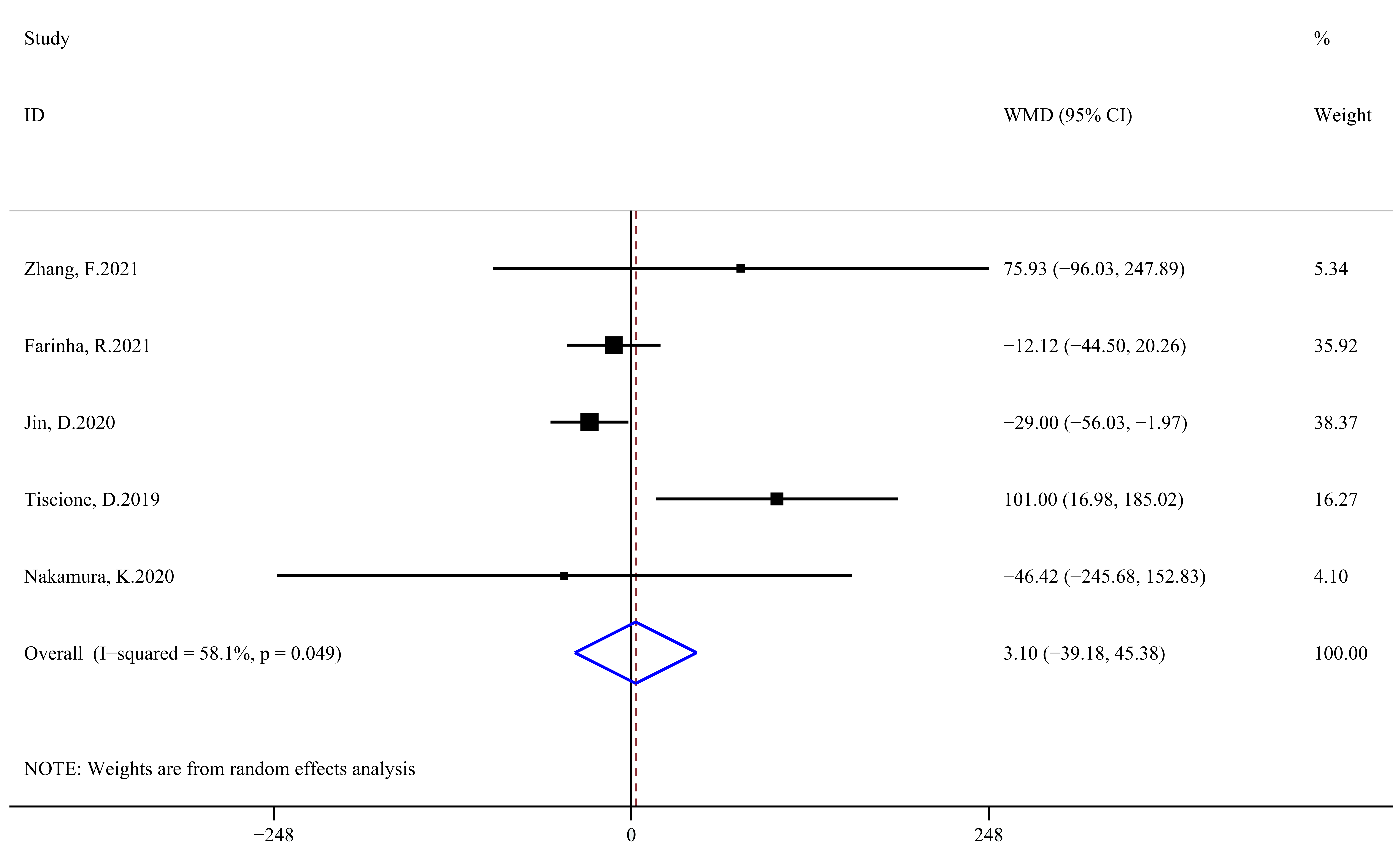


**Figure S2. Sensitivity analysis of estimated glomerular filtration rate**


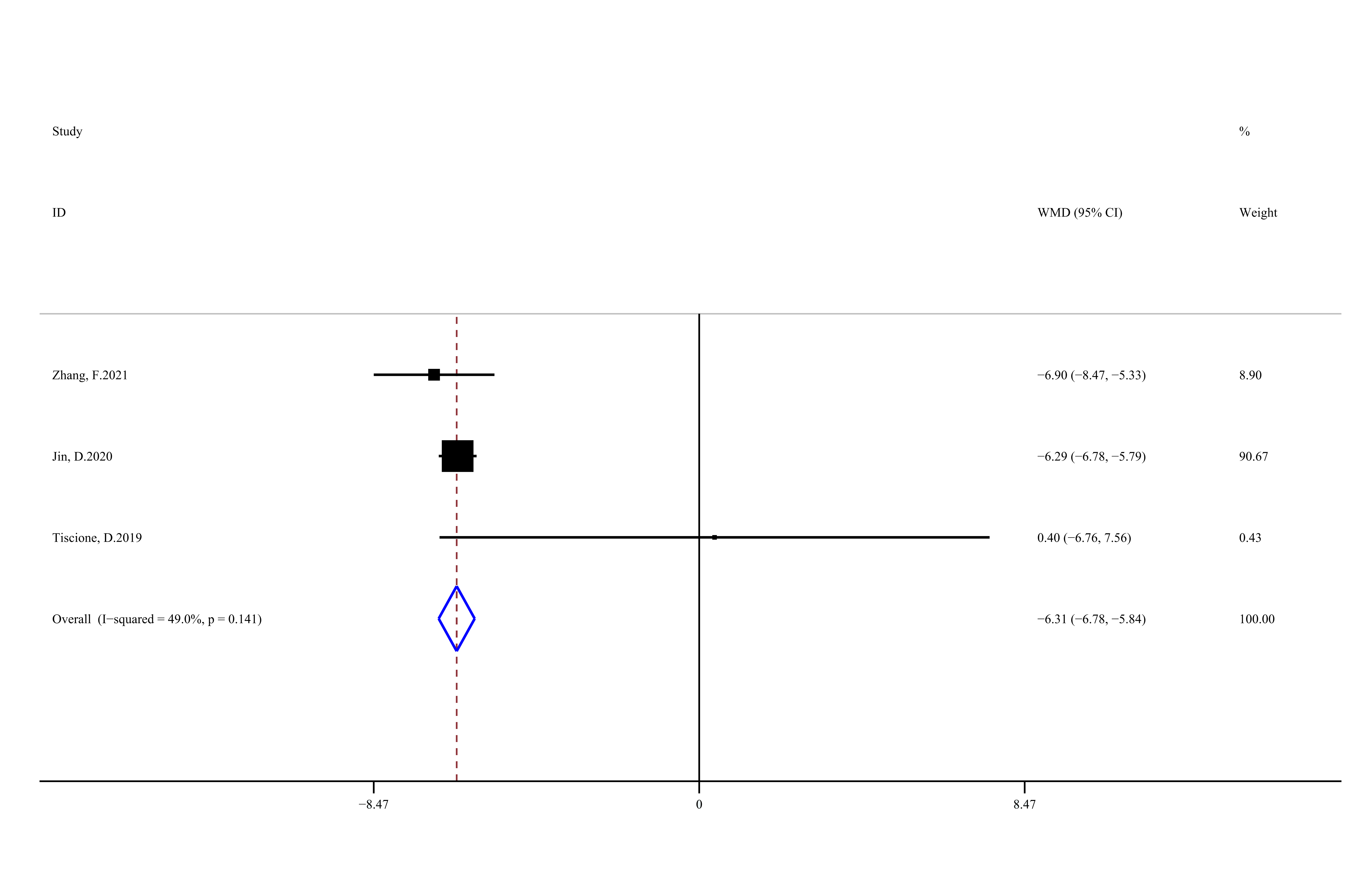


**Figure S3. Sensitivity analysis of operating time**


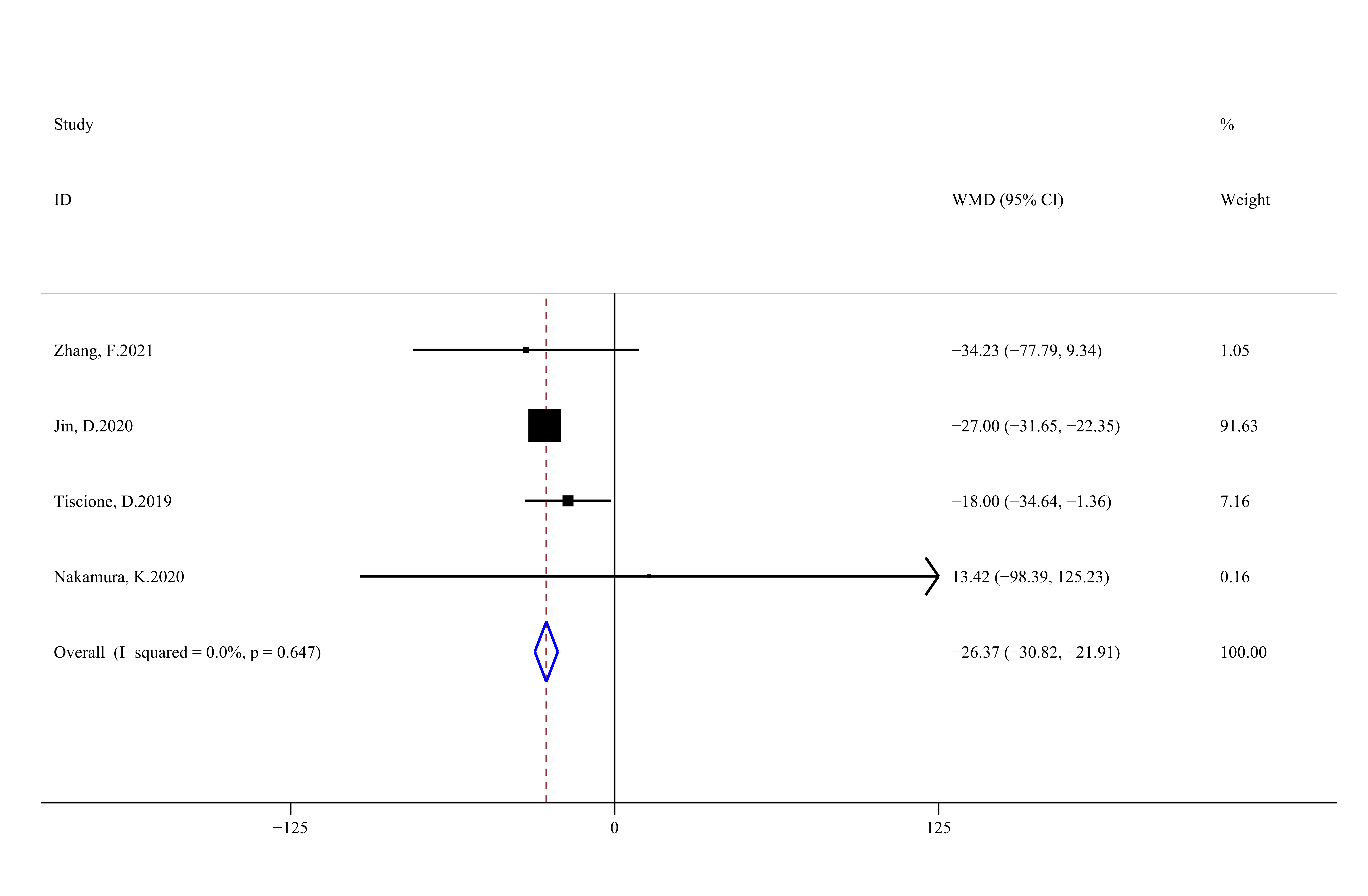


**Figure S4. Sensitivity analysis of blood loss**
