# Supplementary material for: The Outcome of Sutureless in Partial Nephrectomy: A Systematic Review and Meta-Analysis
Source: Biomed Res Int. 2022 Sep 22;2022:5260131. doi: 10.1155/2022/5260131 (PMC9526602; doi:10.1155/2022/5260131)

Sutureless during partial nephrectomy may modify perioperative and renal function outcomes: a systematic review and meta-analysis

Peng Liu[1], Yan Li[1], Benkang Shi[1], Qiujie Zhang[2] and Hu Guo[1]

Correspondence should be addressed to guohu97@sdu.edu.cn

1 Department of urology surgery, Qilu Hospital, College of Medicine, Shandong University, Jinan, China

2 Department of health care, Qilu Hospital, College of Medicine, Shandong University, Jinan, China

Table S1. Search strategy

1. Search strategy on PubMed

(((((((((((((((((((((((((((Robotic Surgical Procedures) OR (Procedure, Robotic Surgical)) OR (Robotic Surgical Procedure)) OR (Robot Surgery)) OR (Robot Surgeries)) OR (Surgery, Robot)) OR (Robot-Assisted Surgery)) OR (Robot Assisted Surgery)) OR (Robot-Assisted Surgeries)) OR (Surgery, Robot-Assisted)) OR (Robot-Enhanced Procedures)) OR (Procedure, Robot-Enhanced)) OR (Robot Enhanced Procedures)) OR (Robot-Enhanced Procedure)) OR (Robotic-Assisted Surgery)) OR (Surgical Procedures, Robotic)) OR (Surgical Procedure, Robotic)) OR (Robotic-Assisted Surgeries)) OR (Surgery, Robotic-Assisted)) OR (Robot-Enhanced Surgery)) OR (Robot Enhanced Surgery)) OR (Robot-Enhanced Surgeries)) OR (Surgery, Robot-Enhanced)) OR (Robotic Assisted Surgery)) OR (Procedures, Robotic Surgical)) OR ((((((((((((((((((((Laparoscopy) OR (Laparoscopies)) OR (Celioscopy)) OR (Celioscopies)) OR (Peritoneoscopy)) OR (Peritoneoscopies)) OR (Surgical Procedures, Laparoscopic)) OR (Laparoscopic Surgical Procedure)) OR (Procedure, Laparoscopic Surgical)) OR (Surgery, Laparoscopic)) OR (Laparoscopic Surgery)) OR (Laparoscopic Surgeries)) OR (Surgeries, Laparoscopic)) OR (Laparoscopic Assisted Surgery)) OR (Surgeries, Laparoscopic Assisted)) OR (Surgery, Laparoscopic Assisted)) OR (Laparoscopic Assisted Surgeries)) OR (Laparoscopic Surgical Procedures)) OR (Procedures, Laparoscopic Surgical)) OR (Surgical Procedure, Laparoscopic))) AND (((((partial nephrectomy) OR (nephron-sparing surgery)) OR (conservative surgery)) OR (enucleation)) OR (enucleoresection))) AND (renorrhaphy OR sutur* OR

2. Search strategy on Embase

('partial nephrectomy'/exp OR 'nephron-sparing surgery':ab,ti OR 'conservative surgery':ab,ti OR enucleation:ab,ti OR enucleoresection:ab,ti) AND ('laparoscopy'/exp OR laparoscopies:ab,ti OR celioscopy:ab,ti OR celioscopies:ab,ti OR peritoneoscopy:ab,ti OR peritoneoscopies:ab,ti OR 'surgical procedures, laparoscopic':ab,ti OR 'laparoscopic surgical procedure':ab,ti OR 'procedure, laparoscopic surgical':ab,ti OR 'procedures, laparoscopic surgical':ab,ti OR 'surgery, laparoscopic':ab,ti OR 'laparoscopic surgical procedures':ab,ti OR 'laparoscopic surgery':ab,ti OR 'laparoscopic surgeries':ab,ti OR 'surgeries, laparoscopic':ab,ti OR 'laparoscopic assisted surgery':ab,ti OR 'laparoscopic assisted surgeries':ab,ti OR 'surgeries, laparoscopic assisted':ab,ti OR 'surgery, laparoscopic assisted':ab,ti OR 'surgical procedure, laparoscopic':ab,ti OR 'robot assisted surgery'/exp OR 'procedure, robotic surgical':ab,ti OR 'procedures, robotic surgical':ab,ti OR 'robotic surgical procedure':ab,ti OR 'surgical procedure, robotic':ab,ti OR 'robot surgery':ab,ti OR 'robot surgeries':ab,ti OR 'surgery, robot':ab,ti OR 'robot-assisted surgery':ab,ti OR 'robot assisted surgery':ab,ti OR 'robot-assisted surgeries':ab,ti OR 'surgery, robot-assisted':ab,ti OR 'robot-enhanced procedures':ab,ti OR 'procedure, robot-enhanced':ab,ti OR 'robot enhanced procedures':ab,ti OR 'robot-enhanced procedure':ab,ti OR 'surgical procedures, robotic':ab,ti OR 'robotic-assisted surgery':ab,ti OR 'robotic assisted surgery':ab,ti OR 'robotic-assisted surgeries':ab,ti OR 'surgery, robotic-assisted':ab,ti OR 'robot-enhanced surgery':ab,ti OR 'robot enhanced surgery':ab,ti OR 'robot-enhanced surgeries':ab,ti OR 'surgery, robot-enhanced':ab,ti) AND ('suture'/exp OR suture:ab,ti OR 'staple, surgical':ab,ti OR 'staples, surgical':ab,ti OR 'surgical staple':ab,ti OR 'surgical staples':ab,ti OR renorrhaphy:ab,ti OR reconstruction:ab,ti OR

	Inclusion criteria	Exclusion criteria
Patient population	Patients >18 years with renal masses, undergoing LPN or RAPN	Patients < 18 years; open partial nephrectomy
Interventions	Sutureless during renorrhaphy	Single-layer suture or other suture techniques during renorrhaphy
Comparator	Double-layer suture (inner and cortical)	Single-layer suture or other suture techniques during renorrhaphy
Outcomes	Renal functional outcomes: decline of eGFR<br>Intra- or perioperative outcomes: OT; WIT; BL; postoperative complication	
Study design	RCT or retrospective study	Case reports, abstract meetings, book chapters, editorials and previous reviews on the topic

Table S2. eligibility criteria

LPN, laparoscopic partial nephrectomy; RAPN, robot-assisted partial nephrectomy; RCT, randomized controlled trail; OT, operating time; WIT, warm ischemia time; EBL, estimated blood loss; eGFR, estimated glomerular filtration rate.

Figure S1. Forest plots of blood loss for sutureless versus double-layer suture partial nephrectomy

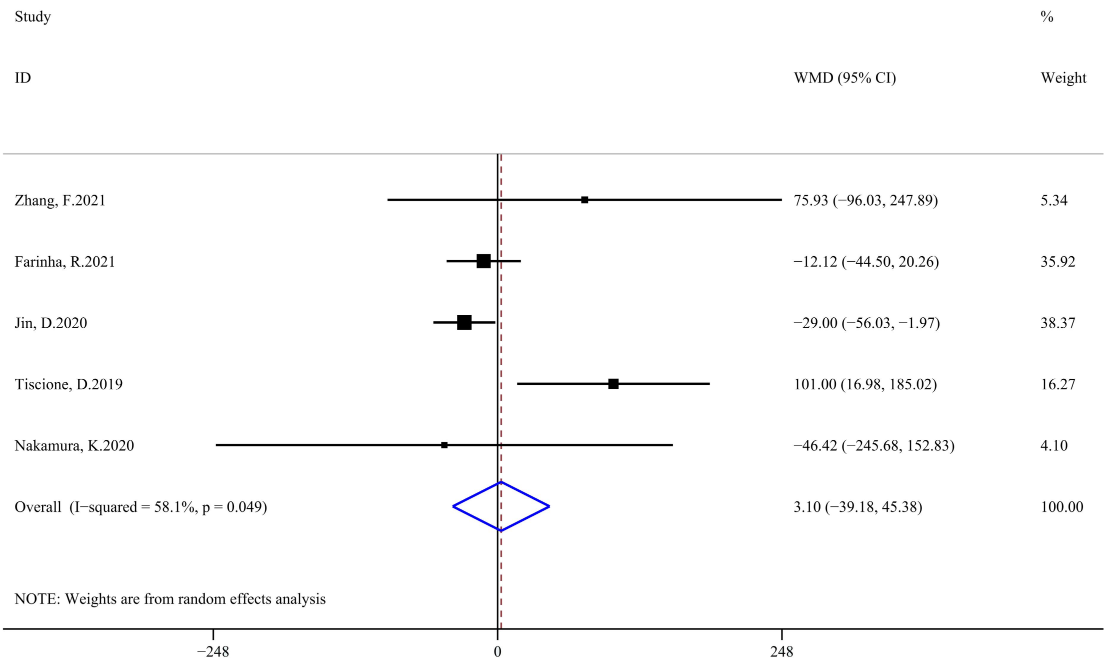

Figure S2. Sensitivity analysis of estimated glomerular filtration rate

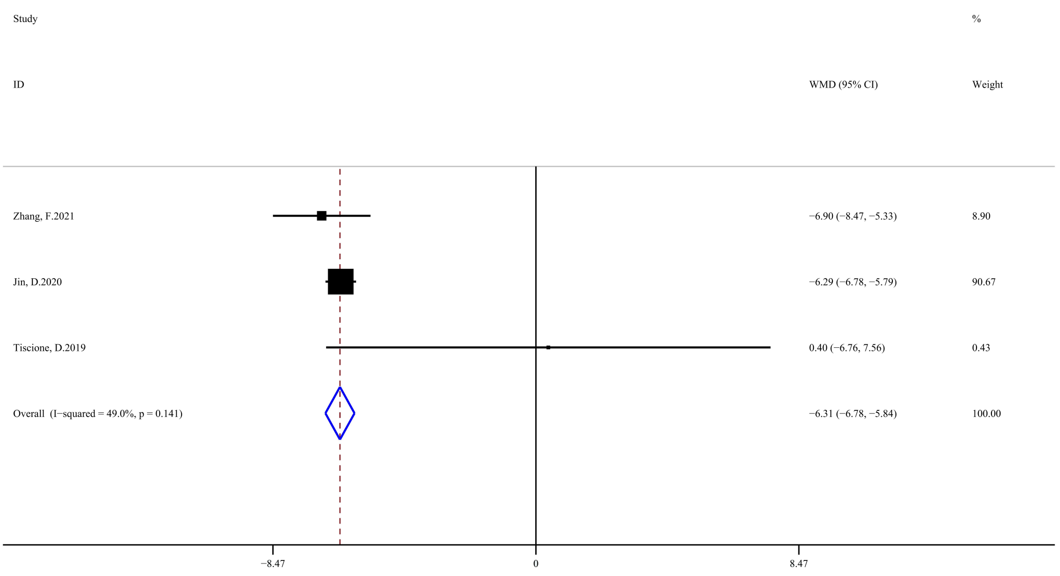

Figure S3. Sensitivity analysis of operating time

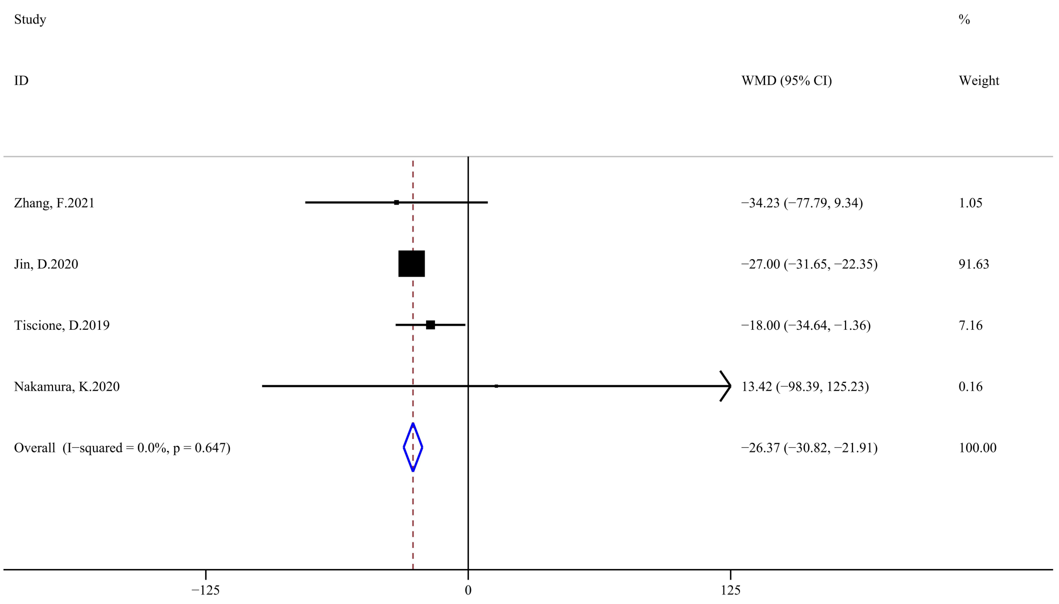

Figure S4. Sensitivity analysis of blood loss

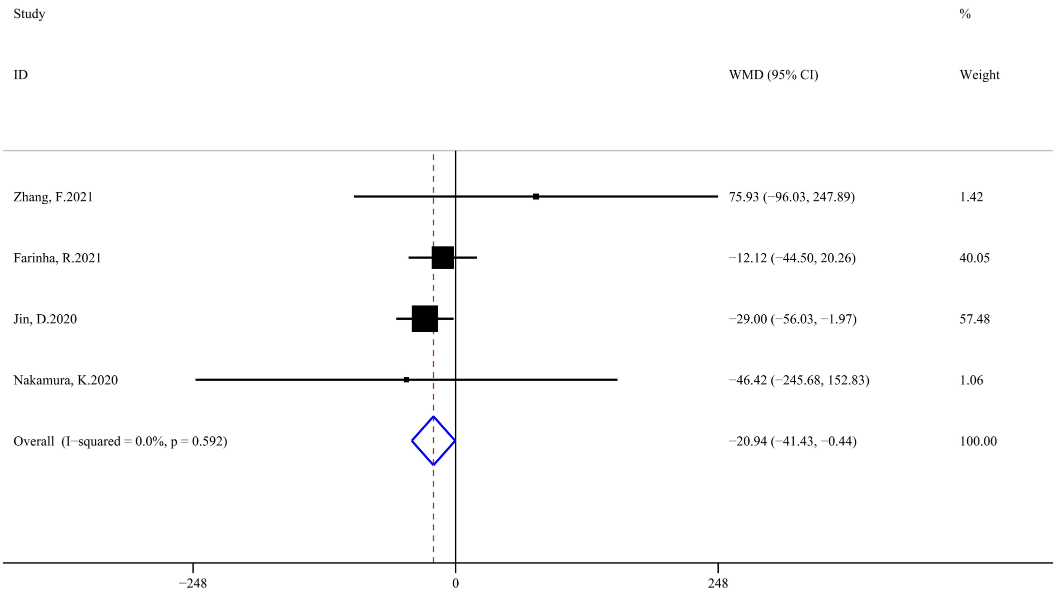

Supplement: Supplementary Materials — Table S1: search strategy. Table S2: eligibility criteria. Figure S1: forest plots of blood loss for sutureless versus double-layer suture partial nephrectomy. Figure S2: sensitivity analysis of estimated glomerular filtration rate. Figure S3: sensitivity analysis of operating time. Figure S4: sensitivity analysis of blood loss. [file 5260131.f1.zip › Supplementary Information.pdf]
